# Addressing ‘hard‐to‐fund’ research questions in bowel research: Exploring the role of decision architecture randomised trials

**DOI:** 10.1111/codi.70589

**Published:** 2026-08-13

**Authors:** Stephen J. Chapman, Carly Bisset, Hayley Fowler, Alice Murray, Jessica Banks, Judit Kovacs, Jim Tiernan, Julie Cornish, Susan Moug, Dale Vimalachandran, James H. Flory, Andrew Vickers, Clare Relton

**Affiliations:** ^1^ Leeds Institute of Medical Research University of Leeds Leeds UK; ^2^ Department of Colorectal Surgery Glasgow Royal Infirmary Glasgow UK; ^3^ Health Education England, North West Manchester UK; ^4^ University College London Hospitals NHS Foundation Trust London UK; ^5^ MRC Unit, The Gambia London School of Hygiene and Tropical Medicine London UK; ^6^ Wolfson Institute of Population Health Queen Mary University London UK; ^7^ Leeds Teaching Hospitals NHS Trust Leeds UK; ^8^ Cardiff and Vale University Health Board Cardiff UK; ^9^ Department of General Surgery Golden Jubilee University National Hospital Clydebank UK; ^10^ Institute of Systems, Molecular and Integrative Biology University of Liverpool Liverpool UK; ^11^ Memorial Sloan Kettering Cancer Centre New York New York USA

**Keywords:** bowel disease, clinical trials, research methods

## Abstract

Patients and health professionals face difficult decisions every day when choosing between standard‐of‐care treatments for the same disease. In bowel disease, some treatments are used interchangeably, but a lack of evidence may exist as to whether one is more effective than another. Randomised controlled trials provide robust evidence in these settings, but they are expensive, time‐consuming and disruptive to usual care. The introduction of electronic health records (EHR) has created unique opportunities to develop efficient trial designs, such as Decision Architecture Randomised Trials (DART). These implement unobtrusive and sometimes indiscernible EHR ‘nudges’, prompting clinicians towards one treatment or another. There is no obligation for clinicians to use the assigned intervention or for the patient to accept it. However, the association between the assignment and the actual treatment received can be used to estimate unbiased treatment effects. We discuss the key features of DART studies and outline the persisting uncertainties to operationalising this study design in clinical practice.

**What does this paper add to the literature?**

Randomised controlled trials (RCT) provide high‐quality evidence but they are expensive, time‐consuming and obstructive to normal care. Some ‘hard‐to‐fund’ questions in bowel disease will never be answered using conventional RCTs. Decision Architecture Randomised Trials (DART) present a possible solution. Here, we describe the concept and outline the persisting uncertainties to operationalising this study design in clinical practice.

## INTRODUCTION

Patients and health professionals face difficult decisions every day when choosing between comparable standard‐of‐care treatments for the same disease. In many situations, multiple treatments can be used interchangeably, but a lack of evidence may exist as to whether one is more effective than another. This creates uncertainty and drives variation in practice which directly affects outcomes for patients and costs for health systems [1]. Randomised controlled trials (RCT) can provide robust evidence in these settings, but they are expensive, time‐consuming and disruptive to usual care. Differences between standard‐of‐care treatments are often small and hence adequately powered RCTs would be very large. Given a limited research budget, funders usually prioritise trials of new treatments over those comparing established treatments. As such, these important but ‘hard‐to‐fund’ questions often remain unanswered. Alternative designs exist but none are ideal; observational studies are inexpensive and deliverable at scale but they are prone to selection bias, precluding a reliable assessment of causal relationships. There is a need for more pragmatic, efficient and deliverable solutions to address these hard‐to‐fund questions in the NHS and other health systems around the world.

## HARD‐TO‐FUND RESEARCH IN BOWEL DISEASE

Bowel disease is common in the United Kingdom (UK), incurring over 1 billion British pounds to the UK National Health Service (NHS) each year. There are over 40,000 new cases of bowel cancer in the UK each year, of which around 60% of patients undergo major surgery [2, 3]. Over 500,000 people in the UK live with inflammatory bowel disease, such as ulcerative colitis and Crohn's disease, which are also common indications for surgery [4]. Other benign bowel diseases add significantly to the burden on NHS resources, including diverticular disease, haemorrhoids, anal fissures, and pelvic floor conditions.

Dilemmas in the choice between multiple standard‐of‐care treatments are engrained in the management of all of these conditions. Examples include choices between

- Diltiazem and Glyceryl trinitrate ointment for the treatment of anal fissure.
- Paracetamol and celecoxib for non‐opioid pain management after colonic resection.
- MoviPrep and Plenvu for effective bowel preparation prior to colonoscopy.
- Surgical clips or monofilament skin closure of specimen extraction sites.
- Qufora and Peristeen irrigation systems for chronic constipation or incontinence.
- Selection of wash (saline or antibiotic saline) in a contaminated surgical field.

Each of these questions is unlikely, or has previously struggled, to attract major research funding for pragmatic RCTs of clinical and cost effectiveness. This is partly a function of sample size since small clinical benefits would require large study populations and substantial financial support. It is also a function of funder and community prioritisation, since questions about new treatments (as opposed to existing standard of care treatments) are often considered to be more important and impactful. Yet, even lower priority questions with smaller expected impact are still relevant to millions of patients with bowel disease in the UK every year. In each of the examples above, and possibly numerous others, it is unclear which option is best and whether a preference towards one or the other may lead to meaningful clinical benefits. This generates missed opportunities to refine existing patient care and reduce healthcare costs.

## DECISION ARCHITECTURE RANDOMISED TRIALS (DART)

The introduction of electronic health records (EHR) in the NHS has created unique opportunities to develop efficient trial designs, such as Decision Architecture Randomised Trials (DART). Decision architecture describes features of an EHR environment which influence choices, such as the format and sequence of options that are presented when an ‘order’ or ‘prescription’ is placed [5]. All else being equal, clinicians will tend to choose the first option listed, or the default option that appears pre‐checked. As such, small changes to how an EHR presents information, such as changing the order in which a medication appears or changing the default option, can be used to ‘nudge’ clinicians towards one treatment or another. DART studies use the decision architecture of EHRs to introduce randomised, unobtrusive, and often non‐apparent nudges into everyday clinical decisions. This enables a randomised assessment of routinely collected clinical outcomes. The association between the nudged assignment and the actual treatment received by patients is used as an instrumental variable to estimate unbiased treatment effects. The strength of a nudge is determined by how often it is overridden by either patients or clinicians, with stronger nudges being overridden less commonly. Nonetheless, even weaker nudges can be used as an instrumental variable provided that sufficient patients are randomised [5].

The scope of research questions that are applicable to a DART study is broad, potentially comprising any standard‐of‐care intervention facilitated using an EHR. This includes electronic prescribing systems for standard‐of‐care medications, electronic requesting systems for clinical investigations such as imaging and blood tests, electronic surgical preference cards for selecting equipment prior to surgery, and electronic referral pathways for cross‐speciality outpatient reviews. To date, early work on DART methods has focussed on standard‐of‐care drug prescribing. For instance, an early proof‐of‐concept analysis in the context of insulin prescribing showed that less‐aggressive bedtime insulin could be used safely for optimising morning glucose control [6]. In breast surgery, a pilot DART study is currently exploring the role of gabapentin as multimodal analgesia (NCT07308717). This randomises patients to a nudge which removes standard‐of‐care gabapentin from template prescription sets, making it more likely that gabapentin will be omitted from the wider strategy of multi‐modal analgesia [7]. The amount of morphine administered to patients is the primary outcome and is recorded routinely using an EHR. An interesting feature of DART studies is that sample size requirements are determined in part by the strength of the nudge. As of this writing, the study has enrolled over 450 patients with a nudge strength of over 80% and is expected to reach target accrual (projected between 1120 and 1580 patients) within 12 months of activation.

Introducing DART studies into healthcare systems presents a number of potential benefits. A key benefit is efficiency, enabling a rapid assessment of multiple standard‐of‐care interventions at reduced cost, with reduced staff burden, and with less disruption to patient care. Since all interventions included in a DART study represent the usual standard‐of‐care, it is expected that research findings would implement seamlessly into practice, minimising the time for impact. Another benefit is the potential for improved research inclusion. The integration of DART studies into routine EHR workflows could overcome social, educational and cultural barriers related to research participation, increasing the representativeness of research to the wider public. This could help to reduce health inequalities within diverse communities. Finally, DART studies would be expected to preserve patient choice and autonomy. Unlike a conventional RCT, there would be no obligation for patients or clinicians to use an assigned treatment, meaning that the usual course of clinical care would continue without disruption. Doing so could increase overall research capacity in the NHS without adding rigid research pathways into usual care.

## CHALLENGES AND UNCERTAINTIES OF DARTs

A number of challenges and uncertainties related to the delivery of DART studies remain which must be addressed before being operationalised in practice.

### Ethical challenges

One of the most critical challenges is ethical oversight since a DART study may not require and may be impossible to conduct with explicit patient consent. The rationale for this is that a DART does not constrain a clinician's ability to prescribe whatever intervention they feel is in the patient's best interest. There is, moreover, no reasonable way to explain to a patient that, although their clinician is able to choose whatever treatment they want, the clinician may be influenced by aspects of an EHR irrespective of whether they are in a trial or not. Nonetheless, ethical uncertainties must be explored critically. Future work will explore these challenges with key stakeholders, including the public, research professionals and research ethics committees.

### Technological challenges

In the UK, it is expected that 95% of NHS hospitals will be using an EHR by 2026, followed by further efforts to improve digital maturity in the years to come [8]. The variation in functionality and cross‐compatibility of EHR systems within and between hospitals is a key uncertainty. While it is already demonstrated that randomisation and routine data collection can take place within the digital environment of an EHR [7], a clear understanding of these capabilities is required across the entire health service. Furthermore, an assessment of how variation in EHR capabilities could be harmonised to enable cross‐compatibility is needed. Future work will map the roll out of EHR systems in the UK, specifically documenting variation in their functionality and their capabilities for delivering research‐specific processes.

### Stakeholder acceptability

While DART studies have the potential to improve efficiency, decrease burden and reduce disruption to care, uncertainties around their acceptability among key stakeholders persist. An appreciation of the public's balance between conventional RCTs and DART studies is required to assess their acceptability about taking part in the future. Similarly, it is important to understand the views and concerns of healthcare professionals whose practice would be the target of electronic nudges during their day‐to‐day activities. An assessment of confidence is also important among research design and delivery professionals, including statisticians, methodologists, delivery teams and regulatory professionals. Future work will explore the views and perspectives of each of these groups, seeking to explore the facilitators and barriers of operationalising DART studies in clinical practice.

## FINAL REMARKS

Bowel disease is a source of great burden for the NHS. Research questions pertaining to existing standard‐of‐care treatments are important but are commonly hard‐to‐fund and slow‐to‐deliver using conventional research methods, such as RCTs. With the widespread introduction of EHRs and an increasingly digital healthcare system, DART studies may represent a unique opportunity to deliver studies of standard‐of‐care interventions more efficiently and without excess burden. Future work will explore challenges relating to DART studies, such as ethical oversight, technological feasibility and stakeholder acceptability. If feasible, DART studies could be operationalised to address previously unanswerable questions in bowel disease research.

## AUTHOR CONTRIBUTIONS

**Hayley Fowler:** Conceptualization; funding acquisition; methodology; writing – review and editing. **Jim Tiernan:** Methodology; writing – review and editing. **Jessica Banks:** Conceptualization; funding acquisition; methodology; writing – review and editing. **Judit Kovacs:** Methodology; writing – review and editing. **Alice Murray:** Conceptualization; funding acquisition; methodology; writing – review and editing. **Carly Bisset:** Conceptualization; funding acquisition; methodology; writing – review and editing. **Julie Cornish:** Methodology; writing – review and editing. **Susan Moug:** Conceptualization; methodology; funding acquisition; writing – review and editing; supervision. **Andrew Vickers:** Conceptualization; methodology; writing – review and editing; supervision. **Stephen J. Chapman:** Conceptualization; investigation; funding acquisition; writing – original draft; methodology; project administration. **James H. Flory:** Conceptualization; methodology; writing – review and editing; supervision. **Dale Vimalachandran:** Conceptualization; funding acquisition; methodology; writing – review and editing; supervision. **Clare Relton:** Methodology; writing – review and editing; supervision.

## FUNDING INFORMATION

This project is funded by Bowel Research UK. SJC is supported by the National Institute for Health and Care Research (NIHR CL‐2022‐02‐05). This work was also supported in part by the National Institutes of Health/National Cancer Institute (NIH/NCI) with a Cancer Center Support Grant to Memorial Sloan Kettering Cancer Center (P30 CA008748) and by Patient Centered Outcomes Research Institute (ME‐2022C1‐26378). The views are those of the authors and not necessarily those of the NHS and other affiliated institutions.

## CONFLICT OF INTEREST STATEMENT

The authors declare no conflicts of interest.

## ETHICS STATEMENT

None.

## CONSENT FOR PUBLICATION

All authors have provided consent for publication.

## Data Availability

Data sharing not applicable to this article as no datasets were generated or analysed during the current study.
